# Metabolic Profiling of *Lactococcus lactis* Under Different Culture Conditions

**DOI:** 10.3390/molecules17078022

**Published:** 2012-07-03

**Authors:** Kamalrul Azlan Azizan, Syarul Nataqain Baharum, Normah Mohd Noor

**Affiliations:** Institute of Systems Biology, Universiti Kebangsaan Malaysia, 43600 Bangi, Selangor, Malaysia

**Keywords:** *Lactococcus lactis* subsp. *cremoris*, metabolic profiling, GC-MS, derivatization

## Abstract

Gas chromatography mass spectrometry (GC-MS) and headspace gas chromatography mass spectrometry (HS/GC-MS) were used to study metabolites produced by *Lactococcus lactis* subsp. *cremoris* MG1363 grown at a temperature of 30 °C with and without agitation at 150 rpm, and at 37 °C without agitation. It was observed that *L. lactis* produced more organic acids under agitation. Primary alcohols, aldehydes, ketones and polyols were identified as the corresponding trimethylsilyl (TMS) derivatives, whereas amino acids and organic acids, including fatty acids, were detected through methyl chloroformate derivatization. HS analysis indicated that branched-chain methyl aldehydes, including 2-methylbutanal, 3-methylbutanal, and 2-methylpropanal are degdradation products of isoleucine, leucine or valine. Multivariate analysis (MVA) using partial least squares discriminant analysis (PLS-DA) revealed the major differences between treatments were due to changes of amino acids and fermentation products.

## 1. Introduction

*Lactococcus lactis* is a facultative anaerobe widely used for dairy production and food fermentation [1,2]. As a member of lactic acid bacteria (LAB), this bacterium typically produces lactate as a major fermentation end product causing medium acidification. The acidification process by *L. lactis* is important as the low pH not only inhibits growth of other microorganism, but also contributes to changes in food texture and biochemical conversions that produce flavors and aromas [3].

The growth of *L. lactis* is strongly influenced by different of stresses, including acidity, temperature and starvation of carbon or nitrogen. As an example, coagulation steps by acidification and heating during cheese manufacturing process affects the growth of *L. lactis* which enhance metabolite changes that influence the texture, taste and smell of the produced cheese [3,4,5,6].

*L. lactis* is auxotrophic for specific amino acids which are also involved in its adaptation to environmental stress [7,8]. Amino acids also make a significant contribution towards the production of metabolites that are associated with flavor and aromatic properties [9]. The relationship between the fermentation end products and changes of amino acids under the influence of temperature and agitation are of great interest because it relates to the central carbon metabolism [10], for better understanding of bacteria adaptation to environmental stress. 

In order to assess the response to environmental stresses, a quantitative and qualitative study of *L. lactis* was carried out using gas chromatography-mass spectrometry (GC-MS) and headspace (HS) analysis. GC-MS requires metabolites to be volatile through derivatization. Common derivatization reagents are trimethylsilyl- (TMS) and methylchloroformate- (MCF) [11]. TMS is effective for sugars, polyols and sulfur-containing compounds, while MCF is preferable for amino acids [12]. TMS allows good fragmentation during electron impact, but the preparation usually requires a long heating step [13]. The application of rapid microwave-assisted (MA) technique in TMS derivatization procedure successfully shortened the heating process [13]. Unlike TMS, which requires completely dry samples, MCF derivatization allows direct derivatization of samples in aqueous solution without heating [11]. 

The aim of this research is to evaluate and determine the extracellular metabolic changes produced by *L. lactis* in response to temperature and agitation. Different derivatization agents was applied to analyze a wide range of metabolites The results were then compared and analyzed using multivariate analysis (MVA) of hierarchical clustering analysis and partial least squares discriminant analysis (PLS-DA). 

## 2. Results and Discussion

### 2.1. Homolactic and Mixed-Acid Fermentation

**Figure 1:**
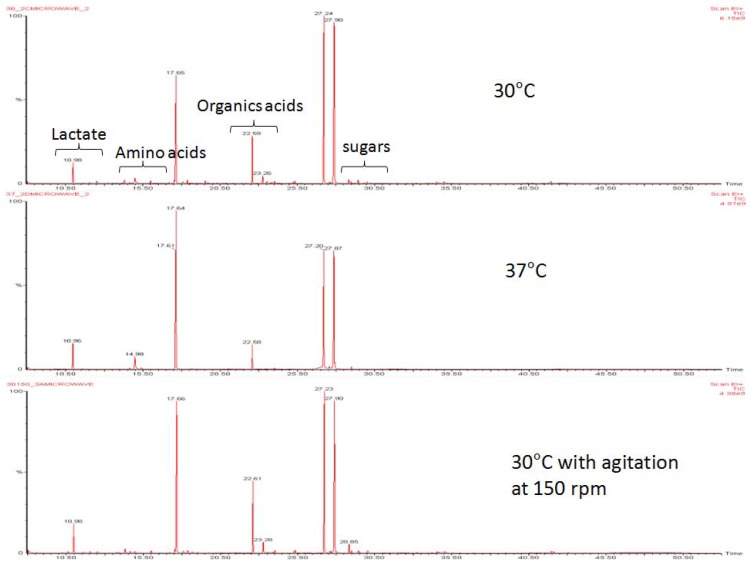
Metabolic profiles of *L. lactis* under growth conditions without agitation at 30 °C, and 37 °C, and 30 °C with agitation. Profiles were obtained using TMS derivatization and measured by GC-MS.

**Figure 2:**
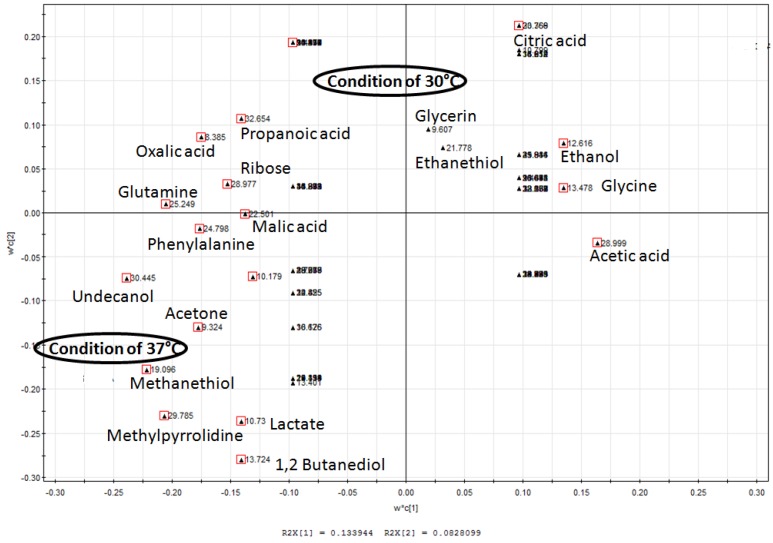
PLS-DA derived loading plot analysis of metabolites profiles. PLS-DA analysis with temperature as the y variable was used to identify the metabolites that distinguished *L. lactis *grown in non-agitated condition of 30 °C and 37 °C. Compounds marked red indicate metabolites with variable importance for projection (VIP) values exceeding 1.

Primary alcohols, aldehydes, ketones, polyol and organic acids of carboxylic acids were present in extracts of *L. lactis* grown at a temperature of 30 °C with and without agitation at 150 rpm, and at 37 °C without agitation (Table 1). Principal component analysis (PCA) revealed that the metabolic profiles at 30 °C and 37 °C without agitation were different (Figure 1). PLS-DA derived loading plot analysis was used to identify metabolites that are significant for the differences (Figure 2). The first two principal components plots showed that fermentation end products of lactate, ethanol and acetate, followed by several organic acids belonging to the tricarboxylic acid (TCA) cycle are detected. Lactate, the major fermentation product of LAB [1], contributed the most under 37 °C growth conditions, compared to ethanol and acetate at 30 °C without agitation. This indicates a shift in metabolism from mixed-acid fermentation at 30 °C to homolactic fermentation at 37 °C. 

**Table 1:** Detected metabolites using TMS derivatization pooled according to conditions of 30 °C, 37 °C and 30 °C with agitation. A.A represents amino acid, F.A represents fatty acid, TCA represents tricarboxylic acid, and O.A represents organic acid.

30 °C	Groups	37 °C	Groups	30 °C agitated	Groups
Alanine	A.A	Proline	A.A	Phenylalanine	A.A
Leucine	A.A	Leucine	A.A	Leucine	A.A
Norleucine	A.A	Phenylalanine	A.A	Serine	A.A
Phenylalanine	A.A	1,3-Propanediol	Alcohol	Acetyl-L-Lysine	A.A
Threonine	A.A	1,2-Butanediol	Alcohol	Aspartic acid	A.A
Glycine	A.A	Propanol	Alcohol	Threonine	A.A
Ethanol	Alcohol	Decanoic acid	F.A	Glutamine	A.A
Propanol	Alcohol	Picolinic acid	F.A	Propanol	Alcohol
Acetone	Ketone	Steric acid	F.A	2-Propanol	Alcohol
2-butanone	Ketone	Hexanoic acid	F.A	1,2-Benzenediol	Alcohol
2-heptanone	Ketone	Hexadecanoic acid	F.A	Isoleucine	Alcohol
Citric acid	TCA	Acetone	Ketone	1,2-Octanediol	Alcohol
Fumaric acid	TCA	2-Pentanone	Ketone	Cadaverine	Biogenic
Acetic acid	O.A	Oxalacetic acid	TCA	Palmitic acid	F.A
Aminobutyric acid	O.A	Lactate	O.A	Hexadecanoic acid	F.A
Hexanoic acid	O.A	3-Hydroxybutyric acid	O.A	2,4-Dihydroxyacetophenone	Ketone
Lactate	O.A	Mercaptoacetic acid	O.A	Heptadecane	Ketone
Mercaptoacetic acid	O.A	Acetic acid	O.A	2-Pentanone	Ketone
Pentadecanoic acid	O.A	Butanoic acid	O.A	Ethanone	Ketone
Propanoic acid	O.A	Octadecanoic acid	O.A	Succinic acid	TCA
Glycerin	Polyol	Pentadecanoic acid	O.A	Malic acid	TCA
Glycerol	Polyol	Hexanoic acid	O.A	Lactate	O.A
Uracil	Pyrimidine	3-Hydroxypyruvic acid	O.A	2-Propenoic acid	O.A
Mannose	Sugar	Ascorbic acid	O.A	Mercaptoacetic acid	O.A
Ribose	Sugar	Acetic acid	O.A	3-Hydroxypyruvic acid	O.A
Xylose	Sugar	Glycerin	Polyol	Ascorbic acid	O.A
Ethanethiol	Sulfur	Glycerol	Polyol	Acetic acid	O.A
Glyceraldehyde		Ribitol	Polyol	Butanoic acid	O.A
		Methanethiol	Sulfurs	Phthalic acid	O.A
				2-Hydroxyisophthalic acid	O.A
				Phosphoric acid	O.A
				Phenylacetic acid	O.A
				Benzoic acid	O.A
				Mandelic acid	O.A
				Oxalic acid	O.A
				Malonic acid	O.A
				Hexanoic acid	O.A
				2-Butenedioic acid	O.A
				Aminomalonic acid	O.A
				Pentanoic acid	O.A

### 2.2. Changes of Amino Acids and Fermentation End Products

**Figure 3:**
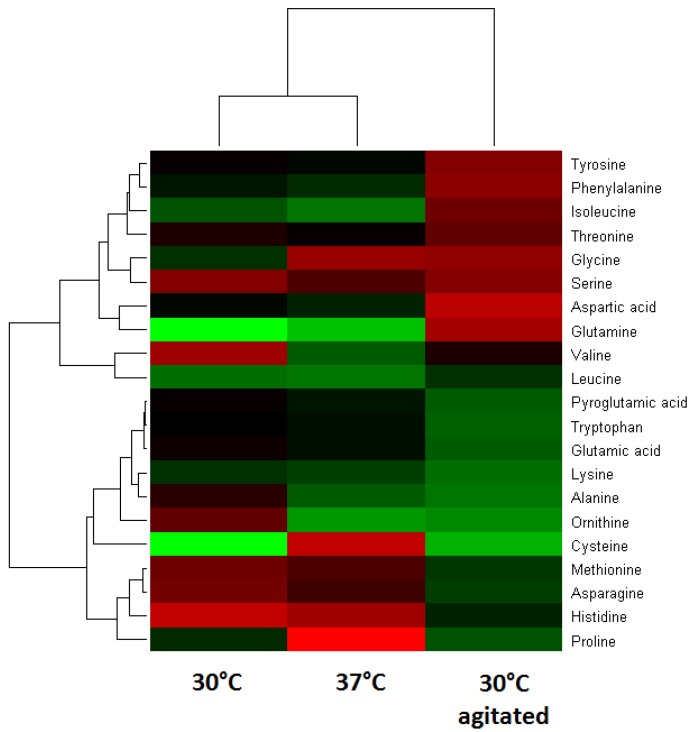
Double hierarchical clustering analysis of detected amino acids from non-agitated conditions of 30 °C and 37 °C, and 30 °C with agitation. Red color indicates relatively high abundance; green represents a relatively low abundance.

Multivariate analysis was performed by hierarchical clustering analysis (HCA) to examine the variations of the amino acids and fermentation end products under stresses of temperature and agitation. As shown in Figure 3, some of the aspartate family (aspartate, glutamine, glycine, serine and threonine) and shikimate-derived amino acids (phenylalanine and tyrosine) are more abundant (red) under the 30 °C agitated condition. In general, aspartate is the precursor to several amino acids including threonine, and isoleucine, while the shikimate pathway via chorismate is essential for the aromatic compounds biosynthesis. Furthermore, greater abundance of phenylalanine and tyrosine particularly under agitated condition suggested a role of phosphoenolpyruvate and erythrose-4-phosphate in the activation of shikimate pathway for the production of these aromatic amino acids.

Under non-agitated conditions, ornithine was more abundant at 30 °C, compared with cysteine at 37 °C. The relationship between larger amounts of ornithine and the fermentation end products ethanol and acetate are likely to be associated with carbon limitation. During exponential phase that lasted 5 to 6 h (data not shown), when the carbon source started to exhaust, there might be a shift toward mixed-acid fermentation. The modification of pyruvate metabolism via pyruvate dehydrogenase activity could explain the production of ethanol and acetate. Furthermore, it was suggested that ornithine is a result from arginine catabolism via the arginine deiminase (ADI) pathway [14]. ADI is responsible for the conversion of arginine into ornithine via citrulline. The activation of ADI was influenced by the carbon starvation and changes of pH. In general, ornithine is not a constituent of casein, thus the presence of ornithine indicates the activation of specific enzyme or pathway to generate the amino acid.

A greater amount of cysteine at 37 °C is consistent to that reported effects at elevated temperature, which affect the incubation time, viable bacterial counts and pH changes [15]. Cysteine plays important roles in protein folding, assembly and stability via the formation of disulfide bonds [16]. The greater amount of lactate at 37 °C suggested a metabolic shift to lactate by pyruvate during carbon limitation. This was indicated by little changes in alanine, which is produced from pyruvate that also influences the production of glycine and serine.

### 2.3. Changes of Amino Acids in Response to Temperature and Agitation

**Figure 4:**
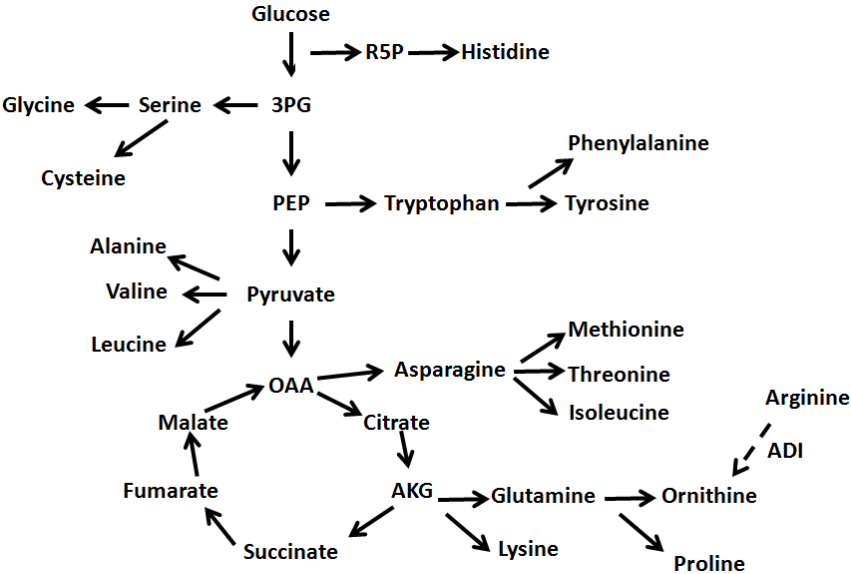
Schematic representation of precursor relationship between amino acids and the central carbon metabolism. R5P represents ribose -5-phosphate, 3PG represents 3-phosphoglycerate and PEP represents phosphoenol pyruvate. OAA represents oxaloacetate and AKG represents 2-oxoglutarate while ADI represents the arginine deiminase.

The HCA in Figure 3 indicated that there are several clusters (horizontal dendrogram) of amino acids changes with two big clusters, larger one (upper cluster) divided into three groups. The lower clusters that grouped serine, glycine, aspartate, tyrosine, phenylalanine and isoleucine and threonine and glutamine indicated that specific pathways were more activated. As shown in Figure 4, the changes of amino acids that were observed at 30 °C with agitation indicated role of 3-phosphoglycerate (3PG) and phosphoenol pyruvate (PEP). The larger amounts of valine and leucine suggest the presence of more pyruvate during agitation stresses while the larger amount of isoleucine would require more threonine via 2-oxobutanoate which is derived from oxaloacetate (OAA). Observation of amino acids changes at 30 °C and 37 °C suggested role of ribose-5-phosphate (R5P) and oxaloacetate (OAA) due to changes of histidine and asparagine, methionine, and threonine. 

### 2.4. Comparison of Metabolites Detected Using TMS, MCF and HS Analysis

TMS is routinely employed in gas chromatography (GC) to increase the chemical volatility and stability of organic metabolites containing active hydrogen [17]. The derivatization is based on the methoximation and silylation enabling the detection of a wide range of metabolite groups, including sugar derivatives, organic acids, fatty acids and amino acids (Table 1). However, the procedure usually requires a long heating treatment and must be carried out under anhydrous conditions [12,13]. The use of microwave- assistance (MA) in TMS derivatization procedure of MSTFA and BSTFA has significantly increased the detection of metabolites while shortening the heating period of methoximation and silylation steps [13,18,19]. The use of microwave irradiation for methoximation and silylation prior to GC-MS analysis has been widely used in biological samples including environmental analysis, herbicides and industrial related processes [19,20,21,22,23,24,25]. In this study, MSTFA which works well with microwave application was used instead of other TMS reagent.

The use of MCF derivatization in GC-MS was first introduced by Husek *et al.* [26]. The derivatization does not require special sample preparation or multiple reaction steps or heating treatment [27]. The alkyl chloroformates based derivatization favors detection of carboxyl group (‑COOH) containing metabolites. As shown in Table 2, detected metabolites are mainly amino acids and organic acids, including metabolites from the citrate cycle and fatty acids. 

**Table 2:** Detected metabolites using MCF derivatization are pooled within groups. Metabolites in bold (*italic*) represent metabolites that are also detected using TMS derivatization.

Organic acids	Amino acids	Fatty acid
2-aminobutyric acid	Alanine	Caprylate
4-aminobutyric acid	***Asparagine***	10,12-Otacedecadienoate
***Caproic acid***	Aspartic acid	
***Citraconate***	***Cysteine***	
***Fumaric acids***	***Glutamic acid***	
***Glutaric acid***	Glycine	
***Glyceric acid***	***Histidine***	
***Hydroxybenzoate***	Isoleucine	
***Itaconic acid***	Leucine	
***Lactate***	***Lysine***	
Malic acid	***Methionine***	
Malonic acid	***Ornithine***	
***Nicotinic acid***	Proline	
***Oxalacetic acid***	Threonine	
***Oxalic acid***	Tryptophan	
***Succinic acid***	Valine	

Comparison of groups of metabolite detected by TMS and MCF (Table 3) indicated that MCF derivatization is best suited for targeted profiling. TMS on the other hand detects a wider range of chemical groups making it as a preferable derivatization method for metabolic profiling experiments.

**Table 3:** Summary of group of metabolites detected by TMS and MCF.

Groups of metabolites	TMS	MCF
Alcohols	Yes	No
Aldehydes	Yes	No
Amines	Yes	No
Amino acids	Yes	Yes
Fatty acids	Yes	Yes
Ketones	Yes	No
Organic acids	Yes	Yes
Sugars	Yes	No

### 2.5. Headspace (HS) Analysis

**Figure 5:**
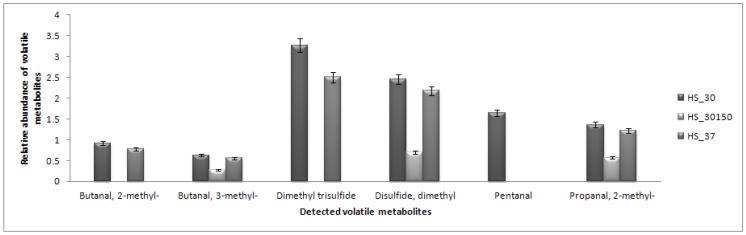
Bar chart representing the relative abundances of detected branched-chain aldehydes using dynamic headspace (HS) coupled to GC-MS according to the three conditions. Condition with agitation showed less production of branched chain aldehydes. HS_30 represents condition of 30 °C, HS_37 represents condition of 37 °C and HS_30150 represents condition with agitation (150 rpm).

Direct analysis using dynamic headspace (HS) coupled to GC-MS was carried out to detect any volatile metabolites that react less with the derivatization reagents. Branched chain methyl aldehydes (2-methylbutanal, 3-methylbutanal and 2-methylpropanal) that are commonly produced by *L. lactis* during cheese manufacturing [9,28,29] were successfully detected, including pentanal and sulfur-based compounds (Figure 5). In brief, aldehydes are the most abundantly produced metabolites by *L. lactis*, especially in the cheese manufacturing process [28,29]. The production of the branched-chain methyl aldehydes was associated with the lower amounts of branched-chain amino acids of leucine, isoleucine and valine, while pentanal was likely from degradation of unsaturated fatty acids [28]. No production of 2-methylbutanal is observed under the agitation condition, although a high isoleucine response was detected under those conditions. 

### 2.6. Partial Least Square Discriminant Analysis

**Figure 6:**
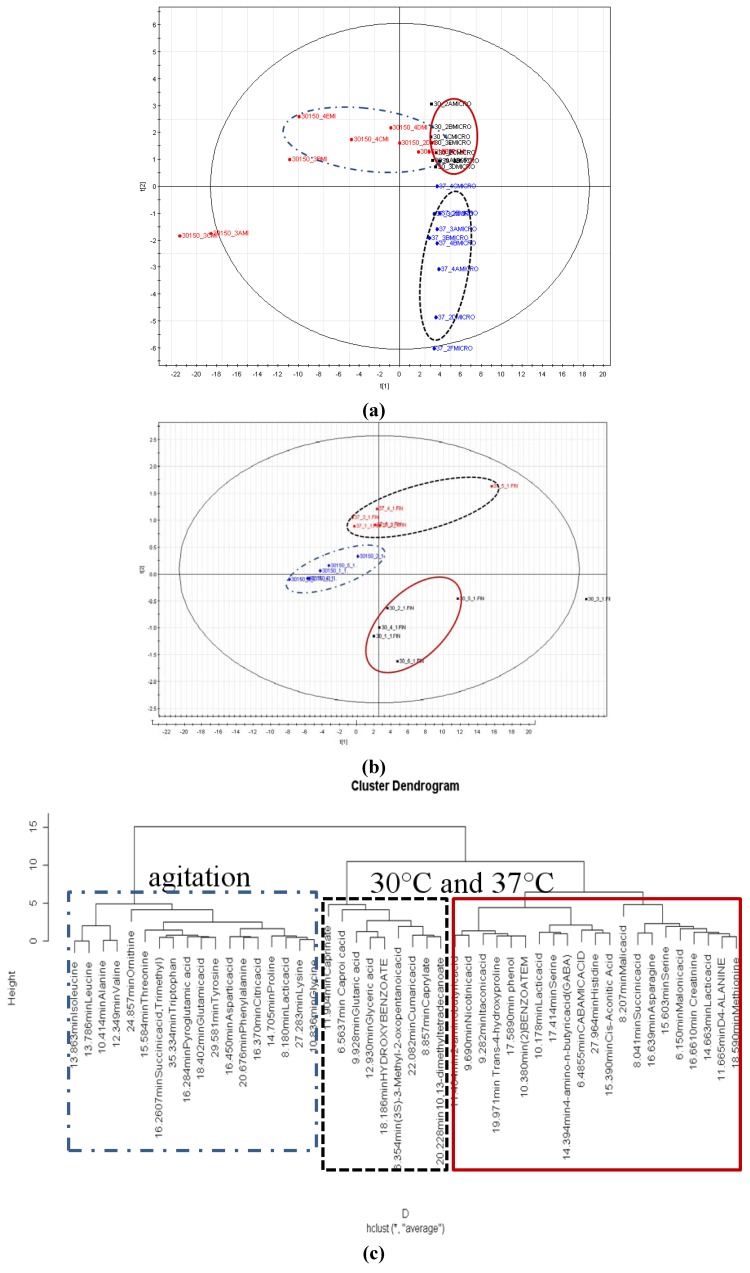
(**a**) PLS-DA score plots by the combination of PC1 and PC2 of TMS derivatised replicates. The ellipses represent confidences of 95% in the Hotelling T2 tests. 30 represents condition of 30 °C, 37 represents condition of 37 °C, 30150 represents condition with agitation (150 rpm). (**b**) PLS-DA score plots by the combination of PC1 and PC2 of MCF derivatised replicates. The ellipses represent confidences of 95% in the Hotelling T2 tests. 30 represents condition of 30 °C, 37 represents condition of 37 °C, 30150 represents condition with agitation (150 rpm). (**c**) Hierarchical clustering analysis of MCF derivatised metabolites according to conditions. Red represents condition of 30 °C, blue represents the condition of agitation and black represents the condition of 37 °C.

Discriminant analysis using supervised PLS-DA for samples derivatised using TMS (Figure 6A) and MCF (Figure 6B) indicated clear separation between the 30 °C, 30 °C with agitation and 37 °C. As MCF derivatization favours the detection of amino acids, the PLS-DA score plot (Figure 6B) is likely to be influenced by the amino acids response. This is supported by the hierarchical clustering analysis (HCA) on the particular metabolites that revealed amino acids to influence the discrimination (Figure 6C).

## 3. Experimental

### 3.1. Chemicals

All chemical reagents used were of analytical grade and purchased from different suppliers (Sigma, Merck, and Burker Corporation). Methylchloroformate (MCF), *N*-methyl-*N*-trimethysilyl trifluoroacetamide (MSTFA) and methoxyamine hydrochloride were obtained from Sigma (St. Louis, MO, USA). Pyridine was purchased from Merck (Whitehouse Station, USA). 

### 3.2. Microbial Cultivation

The plasmid-cured *L. lactis* MG 1363 culture was kindly provided by K. Leenhouts (University of Groningen, Groningen, The Netherlands) and Raha Abd. Rahim (Universiti Putra Malaysia, Serdang, Malaysia). Fermentations were performed under aerobic conditions (O_2_ environment) at 30 °C and 37 °C without agitation and 30 °C with agitation at 150 rpm. The M17 medium used as cultivation medium, (Oxoid Limited, Hampshire, UK) as described by Terzaghi and Sandine [30] contained (in g L^−^^1^): ascorbic acid (0.5), MgSO_4_ (0.25), disodium glycerophosphate (19), tryptone (5), soytone (5), beef extract (5) and yeast extract (2.5). Glucose (0.5%) was added as the carbon source. Samples were collected during the exponential growth (5–6 hours) with OD_600nm _of 1.0.

### 3.3. Growth Estimation

Optical density (OD) at 600 nm was used to provide a measure biomass and constructing growth curve for each of cultivation under the specific conditions. 

### 3.4. Extraction of Extracellular Samples

Approximately 15 mL of fermented culture medium was taken and filtered using a cellulose acetate membrane filter (0.2 µm pore size) to remove the microbial cells. Filtered culture medium was then separated into 1 mL aliquots (n = 5) followed by the addition of dH_2_O (10 mL) and internal standard (0.2 µmol of 10 mmol solution of 2,3,3,3- d4 D,L-alanine) to each of the samples. Samples were then freeze dried under low temperature (−56 °C) and stored at −20 °C. Un-inoculated M17 broth (WT) was also prepared as control.

### 3.5. Sample Derivatization Using TMS

The TMS derivatization method was based on the optimized protocol described by Villas-Boas *et al.* [25] and Rossner *et al.* [31] Briefly, freeze dried samples were resuspended in methoxyamine hydrochloride solution in pyridine (80 µL, 2 g/100 mL), followed by incubation in a domestic microwave (Panasonic NN-K544WF) with multimode irradiation set to 500 W and 50% of exit power for 2.48 min. MSTFA was then added (approximately 80 µL), followed by incubation in the domestic microwave for 3 min, under same conditions as previously mentioned. The final mixed incubation sample was then transferred to a GC-MS vial and analyzed by GC-MS.

### 3.6. Sample Derivatization Using MCF

The MCF derivatization method was based on protocol described by Smart *et al.* [27] and Villas-Boas [25]. Briefly, freeze dried samples were resuspended in NaOH (1M), followed by addition of methanol, pyridine, MCF and chloroform and sodium bicarbonate. The upper aqueous layer in the sample was discarded, and a small portion of anhydrous sodium sulphate was added to dry the remaining reagents. Finally, the dried solution was transferred into a GC-MS vial and analyzed by GC-MS.

### 3.7. Sample Preparation for Headspace Analysis (HS)

Briefly, freeze dried samples were resuspended in 200 µL of dH_2_O and homogenized under temperature of 40 °C before being loaded in headspace apparatus and analyzed using GC-MS.

### 3.8. GC-MS Parameter for Samples Prepared by TMS

The GC-MS parameter used was optimized based on Villas-Boas *et al.* [25] and Rossner *et al.* [31]. GC-MS analysis was performed using the GC-MS Perkin Elmer Turbo Mass Clarus 600 coupled to a quadruple mass selective detector on electron ionization (EI) operated at 70 eV. An aliquot of approximately 1-μL was injected into an Elite-5MS capillary column coated with 5% diphenyl crosslinked and 95% dimethylpolysiloxane (30 m × 0.25 mm i.d. × 0.25 μm thickness) in split mode (50:1). The injection temperature was set to 250 °C, and the ion source temperature was adjusted to 200 °C. The GC method was set from 70 °C to 300 °C with helium gas flow constantly at 1.1 min^−1^. The measurements were made in the full scan mode (*m/z* 45–600).

### 3.9. GC-MS Parameters for Samples Prepared by MCF

The GC-MS parameter used was described by Smart *et al.* [27] and Villas-Boas *et al.* [25]. GC-MS analysis was performed using Agilent GC-MS coupled to a quadruple mass selective detector on electron ionization (EI) operated at 70 eV. An aliquot of approximately 1-μL was injected into J&W 1701 column (30 m × 250 mm i.d. × 0.15 mm) (Folsom, CA). The injection temperature was set to 250 °C, and the ion source temperature was adjusted to 200 °C. The GC method was set from 45 °C to 280 °C with helium gas flow constantly at 1.0 min^−1^. The measurements were made in the scan mode of 38-650 *m/z* at 1.47 scan per sec.

### 3.10. GC-MS Parameters for Headspace Analysis (HS)

A Perkin Elmer TurboMatrix Headspace Sampler 40XL connected to a GC-MS Perkin Elmer Turbo Mass Clarus 600 was used for volatile compounds analysis. A minimal of three duplicates were subjected to helium purge and concentrated in a Tenax trap, kept at 40 °C. Line temperature was adjusted to 180 °C, while helium flow was set at 40 mL/min. Sample temperature was 80 °C, with dry purge time was 1min and desorbed temperature was 200 °C. Desorbed time was 1min, and injection port temperature was set to 200 °C. GC-MS analysis was performed using electron ionization (EI) operated at 70 eV. An aliquot of approximately 1 μL was injected into an Elite-5MS capillary column coated with 5% diphenyl crosslinked and 95% dimethylpolysiloxane (30 m × 0.25 mm i.d. × 0.25 μm thickness) in split mode (50:1). The injection temperature was set to 250 °C, and the ion source temperature was adjusted to 200 °C. The GC method was set from 45 °C to 220 °C with helium gas flow constantly at 1.0 mL min^−1^. The measurements were made in the scan mode of *m/z* 33–220.

### 3.11. Data Analysis and Validation

The general approach used for data analysis and validation was performed according to Smart *et al.* [27] and Villas-Boas *et al.* [25]. In summary, detected metabolites were identified using in-house TMS and MCF MS library of derivatised pure standard developed by Villas-Boas. For peaks that have not been identified was identified using NIST mass spectral database library (NIST 2008) with cut-off similarity of 90%. The value of height of the peak was used to represent the detected metabolites. The values were firstly normalized by total sum of GC height and internal standard followed by log transformed. One-way Analysis of Variance (ANOVA) was used to statistically validate the values followed by comparison using Fisher’s least significant difference (LSD) method with significance levels of *P *< 0.05, *P *< 0.01 and *P *< 0.001 [32]. Visualization of the clean, validated data was then carried out using Principal Component Analysis (PCA) and PLS-DA of Simca-P+ version 12.0 (Umetrics AB, Ume, Sweden) for group classification and discrimination analysis with Q^2^ value > 50%. The heatmap with hierarchical clustering analysis was performed using R script (http://www.r-project.org) with the ward method. 

## 4. Conclusions

GC-MS and HS analysis on the metabolites produced by *L. lactis* in response to temperature and agitation contribute to the understanding of metabolic changes during environmental stresses. Meanwhile, the use of TMS derivatization provides a wider range of metabolites detection compared to MCF derivatization which specifically targets amino acids. The PLS-DA derived analyses indicate a strong relationship between fermentation end products of lactate, ethanol, acetate and amino acid changes according to temperature. Finally, these specific responses can serve as optimization factors useful for dairy food production which uses *L. lactis* as starter culture. 

## References

[1] Losiane E.G., Sylvain M. (2011). Bacteriophages of lactic acid bacteria and their impact on milk fermentations. Microb. Cell Fact..

[2] Bermúdez-Humarán L.G., Kharrat P., Chatel J.-M., Langella P. (2011). *Lactococci* and *lactobacilli* as muscosal delivery vectors for therapeutic proteins and DNA vaccines. Microb. Cell Fact..

[3] Mireille Y., Chistophe G., Emilie C., Gaelle B., Veronique M. (2011). The initial efficiency of the proteolytic systems of *Lactococcus lactis* strains determines their responses to a cheese environment. Int. Dairy J..

[4] Guimont C. (2002). Change of free amino acids in M17 medium after growth of *Streptococcus thermophilus* and identification of a glutamine transport ATP-binding protein. Int. Dairy J..

[5] Amel T., Nassra D., Maryse L., Denis R., Gisele L. (2011). Comparative transcriptome analysis of *Lactococcus lactis* subsp. *cremoris* strains under conditions simulating Cheddar cheese manufacture.

[6] Ziadi M., Bergot G., Courtin P., Chambellon E., Hamdi M., Yvon M. (2010). Amino acid catabolism by *Lactococcus lactis* during milk fermentation. Int. Dairy J..

[7] Ayad E.H.E., Verheul A., de Jong C., Wouters J.T.M., Smit G. (1999). Flavour forming abilities and amino acid requirements of *Lactococcus lactis* strains isolated from artisanal and non-dairy origin. Int. Dairy J..

[8] Clementine D., Emma R., Christophe G., Pascal L., Veronique M., Muriel C.B. (2011). Investigation of the adaptation of *Lactococcus lactis* to isoleucine starvation integrating dynamic transcriptome and proteome information. Microb. Cell Fact..

[9] Grade S., Carbonell M., Fernandez-grarcia E., Medina M., Nunez M. (2002). Volatile compounds in Hispanico Cheese manufactured using a mesophilic starter a thermophilic starter and bacteriocin-producing *Lactococcus lactis* subps. *cremoris* INIA415. J. Agric. Food Chem..

[10] Zhu D., Zhou X., Jin Y.Y. (2010). Metabolome profiling reveals adaptive evolution of S. Cerevisiae during repeated vacuum fermentations. Metabolomics.

[11] Villas-Boas S.G., Delicado S.G., Akesson M., Nielsen J. (2003). Simultaneous analysis of amino and nonamino organic acids as methyl chloroformate derivatives using gas chromatography-mass spectrometry. Anal. Biochem..

[12] Cheng W.P., Yang X.Y., Hegeman A.D., Gray W.M., Cohen J.D. (2010). Microscale analysis of amino acids using gas chromatography-mass spectrometry after methyl chloroformate derivatization. J. Chromatogr. B.

[13] Liebeke M., Wunder A., Lalk M. (2009). A rapid microwave-assisted derivatization of bacterial metabolome samples for gas chromatography/mass spectrometry analysis. Anal. Biochem..

[14] Urancken G., Rimaux T., Wouters D., Leny F., De Vuyst L. (2009). The arginine deiminase pathway of *L. fermentum* IMDO 13010 response to growth under stress conditions of both temperature and salt. Food Microbiol..

[15] Dong X., Quinn P.J., Wang X. (2011). Metabolic engineering of *Escherichia coli glutamicum* for the production of L- threonine. Biotechnol. Adv..

[16] Brice S., Patrice P., Dusko S.E., Pierre R., Eric G. (2005). Sulfur amino acid metabolism and its control in *L. lactis* IL1403. J. Bacteriol..

[17] Beale D.J., Dunn M.S., Morrison P.D., Porter N.A., Marlow D.R. (2012). Characterisation of bulk water samples from copper pipes undergoing microbially influenced corrosion by diagnostic metabolomic profiling. Corros. Sci..

[18] Kouremenos K.A., Harynuk J.J., Winniford W.L., Morrison P.D., Marriott P.J. (2010). One-pot microwave derivatization of target compounds relevent to metabolomics with comprehensive two-dimensional gas chromatography. J. Chromatogr. B.

[19] Chu T.Y., Chang C.H., Liao Y.C., Chen Y.C. (2001). Microwave-accelerated derivatization processes for the determination of phenolic acids by gas chromatography-mass spectrometry. Talanta.

[20] Ranz A., Maier E., Motter H., Lankmayr E. (2008). Extraction and derivatization of polar herbicides for GC-MS analyses. J. Sep. Sci..

[21] Bowden J.A., Dominic M.C., Whitney L.S., Diana C.M.M., Timothy J.G., Richard A.Y. (2009). Enhanced Analysis of Steroids by Gas Chromatography/Mass Spectrometry using Microwave-Accelerated Derivatization. Anal. Chem..

[22] Beale D.J., Michael S.D., Donavan M. (2010). Application of GC-MS metabolic profiling to “blue-green water” from microbial influenced corrosion in copper pipes. Corros. Sci..

[23] Sandra L.S., Markus D., Oliver Kappe C. (2010). Microwave-assisted derivatization procedures for gas chromatography/mass spectrometry analysis. Mol. Divers..

[24] Ruiz-Matute A.I., Hernandez-Hernandez O., Rodriguez-Sanchez S., Sanz M.L., Martinez-Castro I. (2011). Derivatization of carbohydrates for GC and GC-MS analyses. J. Chromatogr. B.

[25] Villas-Boas S.G., Noel S., Lane G.A., Attwood G., Cookson A. (2006). Extracellular metabolomics: A metabolic footprinting approach to assess fiber degradation in complex media. Anal. Biochem..

[26] Husek P., Matucha P., Vrankova A., Simek P. (2003). Simple plasma work-up for a fast chromatographic analysis of homocysteine, cysteine, methionine and aromatic amino acids. J. Chromatogr. B.

[27] Smart K.F., Aggio R.M.B., Houtte J.R.V., Villas-Boas S.G. (2010). Analytical platform for metabolome analysis of microbial cells using methyl chloroformate derivatization followed by gas chromatography-mass spectrometry. Nat. Protocol.

[28] Yvon M., Rijnen L. (2001). Cheese flavour formation by amino acid catabolism. Int. Dairy J..

[29] Marilley L., Casey M.G. (2004). Flavours of cheese products: Metabolic pathways analytical tool and identification of producing strains. Int. J. Food Microbiol..

[30] Terzaghi B.E., Sandine W.E. (1975). Improved medium for lactic streptococci and their bacteriophages. App. Microbiol..

[31] Roessner U., Wagner C., Kopka J., Trethewey R.N., Willmitzer L. (2010). Simultaneous analysis of metabolites in potato tuber by gas chromatography-mass spectrometry. Plant J..

[32] Xia J., Psychogios N., Young N., Wishart D.S. (2009). MetaboAnalyst: A web server for metabolomic data analysis and interpretation. Nucleic Acids Res..

